# Deubiquitinase USP8 regulates the spindle assembly checkpoint in oocytes

**DOI:** 10.1126/sciadv.aeb2345

**Published:** 2026-03-13

**Authors:** Changyin Zhou (周长银), Xue Zhang (张雪), Genlu Xu (许根露), Hui Wang (王慧), Yuting Ran (冉宇婷), Ang Li (李昂), Qing-Yuan Sun (孙青原), Xiang-Hong Ou (欧湘红)

**Affiliations:** ^1^ Guangzhou Key Laboratory of Metabolic Diseases and Reproductive Health, Guangdong-Hong Kong Metabolism & Reproduction Joint Laboratory, Reproductive Medicine Center, The Affiliated Guangdong Second Provincial General Hospital of Jinan University, Guangzhou 510317, China.; ^2^ Key Laboratory of Regenerative Medicine of Ministry of Education, Jinan University, Guangzhou 510632, China.; ^3^ Department of Developmental Biology, School of Basic Medical Sciences, Southern Medical University, Guangzhou 510515, China.

## Abstract

The spindle assembly checkpoint (SAC) is vital for preventing oocyte aneuploidy, a leading cause of female infertility, miscarriages, and trisomy syndromes. However, whether deubiquitination participates in SAC regulation remains unknown. Here, we reveal that the deubiquitinase USP8 acts as a SAC regulator to prevent aneuploid egg formation. Mechanistically, depletion of USP8 inactivates the SAC, accelerates meiotic progression, and causes abnormal spindle assembly and chromosome alignment, ultimately leading to aneuploidy. Intriguingly, we identify USP8 in oocytes as a previously unidentified interaction partner of BUB3, a key component of the SAC, and demonstrate that USP8 stabilizes BUB3 through its deubiquitinating activity. Moreover, exogenous BUB3 rescues the defects observed in USP8-depleted oocytes. Together, our findings not only clarify that deubiquitination participates in regulating the SAC in oocytes but also uncover a unique role for USP8 in controlling the SAC via its interaction with BUB3.

## Teaser

Deubiquitination regulates the spindle assembly checkpoint to ensure oocyte ploidy crucial for preventing trisomy and miscarriage.

## INTRODUCTION

The regulation of protein homeostasis by ubiquitination and deubiquitination, which is controlled by ubiquitin E3 ligases and deubiquitinating enzymes (DUBs), is crucial for modulating a wide range of biological processes (*1*, *2*). E3 ubiquitin ligases identify particular substrates and either directly catalyze or facilitate the transfer of ubiquitin to lysine residues on these substrates. In contrast, DUBs can counteract this process by removing ubiquitin from the substrate proteins (*3*). The process of protein deubiquitination is typically catalyzed by ubiquitin-specific proteases (USPs) from the deubiquitinase family (*4*, *5*).

USP8 belongs to the family of USPs and frequently deconjugates ubiquitin from its substrates to regulate the fates of cellular proteins (*6*). In *Drosophila*, deletion of the *Usp8* deubiquitinase gene can convert incomplete divisions of germline cells into complete divisions (*7*). It has recently been found that, in the endoreplicating *Drosophila* salivary gland and Bombyx silk gland, the depletion of USP8 leads to endoreplication arrest and a reduction in gland size (*8*). The transcription of CLK/CYC is inhibited by USP8-mediated deubiquitination of CLOCK in *Drosophila* (*9*). USP8 is recruited to Rab5-positive vesicles via Rabex5 and acts through both the endosomal dissociation of Rabex5 and the recruitment of SAND-1/Mon1 to facilitate endosome maturation in *Caenorhabditis elegans* (*10*).

The absence of USP8 leads to mouse embryonic lethality, whereas its conditional knockout in adults triggers fatal liver failure (*11*). USP8 is involved in acrosome formation in mice (*12*–*14*). USP8 amplifies cGAS-driven type I interferonopathies by deubiquitinating DDX3X (*15*). USP8 also plays a crucial role in the development and maintenance of T cell homeostasis (*16*). Inhibiting USP8 or AKT can significantly reduce MDA5-induced autoimmunity (*17*). Usp8 regulates the clearance of α-synuclein and alters its toxicity in Lewy body disease (*18*). Recent findings show that inhibiting USP8 not only makes tumor cells more susceptible to ferroptosis (*19*–*22*) but also demonstrates great potential in cancer therapy (*23*–*33*). USP8 mutations can lead to Cushing’s disease, which is caused by corticotroph adenomas of the pituitary (*34*–*36*). The deubiquitination of BRIT1 by USP8 regulates the DNA double-strand break response in the U2OS cell line (*37*). USP8 associates with HD-PTP to drive epidermal growth factor receptor (EGFR) sorting to intralumenal vesicles (*38*). USP8 enhances SMO signaling through inhibition of its ubiquitination and alteration of its subcellular distribution (*39*). USP8 also exerts a significant influence on the regulation of autophagy (*40*–*43*).

The spindle assembly checkpoint (SAC) monitors the proper attachment of spindle microtubules to kinetochores, licensing oocytes to enter anaphase and ensuring accurate bivalent segregation (*44*, *45*). Aberrant SAC activity frequently leads to aneuploidy, and mitotic defects can promote oncogenic transformation. Errors during oocyte meiosis are associated with trisomy syndrome or miscarriage (*44*, *46*–*49*). A hallmark of the SAC is its inhibition of the E3 ubiquitin ligase complex APC/C^CDC20^ to block anaphase onset, thereby safeguarding oocyte euploidy through coordinated regulation (*44*, *45*, *50*, *51*). Whether deubiquitination also participate in SAC function to maintain oocyte euploidy remains unknown.

In this study, we reveal the unprecedented role of deubiquitinase USP8 in regulating the SAC to maintain oocyte euploidy by demonstrating that it stabilizes its previously unidentified substrate BUB3 via deubiquitination, thereby establishing deubiquitination as a critical regulatory mechanism alongside ubiquitination for the SAC.

## RESULTS

### Subcellular localization and expression of USP8 during oocyte meiosis

**Fig. 1.**
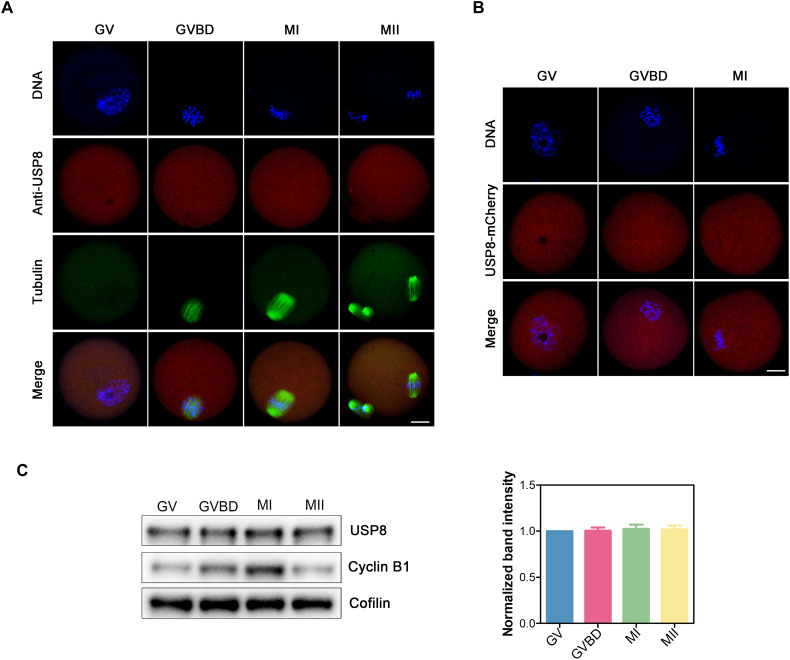
Expression dynamics of USP8 in mouse meiotic oocytes. (**A**) Representative images of USP8 localization in oocytes. Mouse oocytes were immunostained with USP8 and α-tubulin antibodies. Scale bar, 20 μm. (**B**) Representative images of USP8-mCherry localization during mouse oocyte meiosis. Mouse oocytes were microinjected with USP8-mCherry mRNA at the GV stage, maintained for 2 hours in 50 μM IBMX before being washed into IBMX-free medium to allow development to GVBD and MI stages, followed by DNA staining with Hoechst. Scale bar, 20 μm. (**C**) Protein levels of USP8 during oocyte meiosis were examined at the GV, GVBD, MI, and MII stages using immunoblotting analysis. The blots were probed with anti-USP8 antibody, anti–Cyclin B1 antibody, and anti-Cofilin antibody, respectively. The band intensity of USP8 was normalized with that of Cofilin. The GV-stage oocyte is characterized by a distinct GV, enabling clear morphological identification. The GVBD stage is defined by the recent breakdown and disappearance of the GV, whereas the MII stage can be identified by the presence of a prominent first polar body. Meanwhile, Cyclin B1 protein levels are dynamically regulated during oocyte meiotic maturation, gradually increasing from the GV to MI stage and subsequently decreasing at the MII stage compared to MI. Thus, these Cyclin B1 expression patterns can serve as supplementary indicators for determining oocyte meiotic maturation stages.

To investigate the potential role of USP8 during meiotic maturation, we first examined its subcellular localization in mouse oocytes. Immunofluorescence and confocal imaging revealed that USP8 is broadly distributed in oocytes and shows similar USP8 fluorescence signal intensities across all stages of oocyte maturation (Fig. 1A). The markedly weaker USP8 immunostaining signal in knockdown oocytes compared to controls serves as a critical validation of the antibody’s specificity (fig. S1C). This localization was confirmed by the localization of exogenous USP8–mCherry expressed in oocytes (Fig. 1B). Furthermore, following microinjection of either mCherry or USP8-mCherry mRNA into oocytes, the USP8-mCherry group exhibited a stronger USP8 signal compared to the mCherry-only control (fig. S1D), and a distinct band corresponding to the USP8-mCherry fusion protein was detected (fig. S1E). We subsequently evaluated the protein expression of USP8 during oocyte meiosis. Oocytes from germinal vesicle (GV), germinal vesicle breakdown (GVBD), metaphase I (MI), and metaphase II (MII) stages were analyzed by immunoblotting with an anti-USP8 antibody, revealing that USP8 is constantly expressed during oocyte meiotic maturation (Fig. 1C).

### Depletion of USP8 accelerates oocyte meiotic progression

**Fig. 2.**
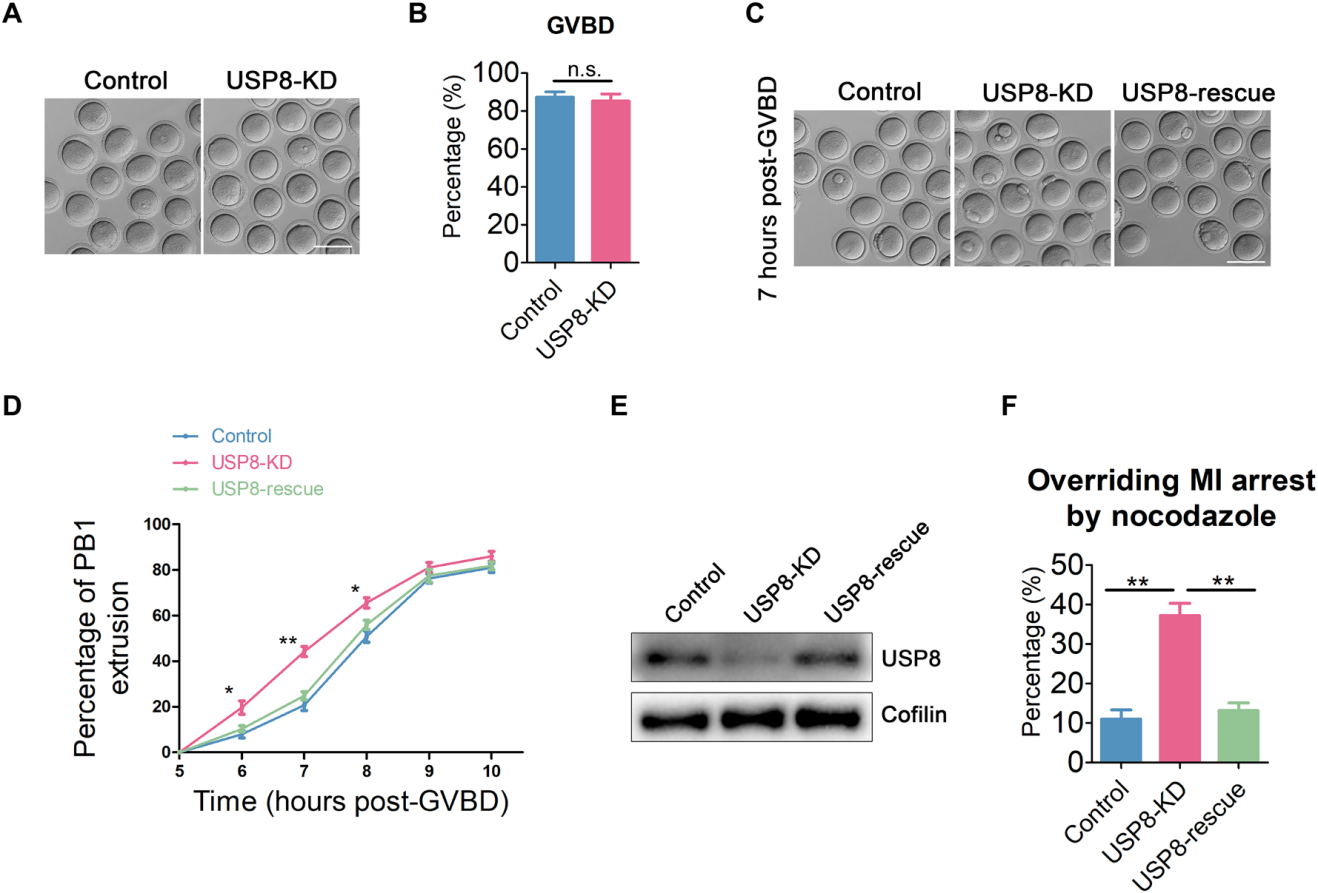
Effect of USP8 depletion on meiotic progression in mouse oocytes. (**A**) Representative images of the occurrence of GVBD in control and USP8-KD. Scale bar, 100 μm. (**B**) The incidences of GVBD were quantified in control (*n* = 118) and USP8-KD (*n* = 108) oocytes. (**C**) Representative images of PBE in control, USP8-KD, and USP8-rescued oocytes at the time point of 7 hours post-GVBD. Scale bar, 100 μm. (**D**) Quantitative analysis of PBE rates was shown in control (*n* = 126), USP8-KD (*n* = 122), and USP8-rescue (*n* = 116) oocytes at consecutive time points of post-GVBD. (**E**) Protein levels of USP8 were assessed by immunoblots in control, USP8-KD, and USP8-rescue oocytes. The blots were probed with USP8 and Cofilin antibodies. (**F**) The proportion of oocytes overriding MI arrest following nocodazole treatment was recorded in control (*n* = 101), USP8-KD (*n* = 102), and USP8-rescue (*n* = 99) oocytes. Oocytes injected with the indicated siRNA and/or mRNA were cultured with 400 nM nocodazole from 4 hours post-GVBD, and the PBE rate was scored at 10 hours post-GVBD. Data were presented as the mean value (means ± SEM) of at least three independent experiments. *P < 0.05; **P < 0.01; n.s., not significant.

To examine USP8 function in oocyte meiosis, loss-of-function studies using gene-specific small interfering RNA (siRNA) were conducted, leading to effective and specific knockdown of USP8 at both mRNA and protein levels (fig. S1, A and B). To evaluate USP8 loss on meiotic progression, we monitored the occurrence of GVBD and polar body extrusion (PBE)—key meiotic milestones. Our results indicated that USP8 depletion had no marked effect on GVBD (Fig. 2, A and B). However, unexpectedly, USP8-depleted oocytes exhibited a notably elevated PBE rate at 6 to 8 hours after GVBD compared with controls (Fig. 2, C and D). To exclude potential off-target effects of the siRNA, exogenous USP8 was introduced into USP8-depleted oocytes to assess meiotic progression. As expected, the rescued oocytes displayed a PBE frequency comparable to that of the control group (Fig. 2, C and D). Successful and specific knockdown and subsequent restoration of USP8 expression were confirmed by Western blot analysis (Fig. 2E).

The premature PBE phenotype suggests a possible defect in SAC activity (*45*, *51*). To further explore the role of USP8 in SAC regulation, we tested whether USP8-deficient oocytes could escape the MI arrest triggered by nocodazole. Nocodazole induces partial microtubule depolymerization and spindle instability, thereby activating SAC and causing oocytes to arrest at MI, blocking anaphase onset. If SAC function is impaired, however, a substantial proportion of oocytes will bypass MI arrest and undergo PBE. Accordingly, we observed that USP8-depleted oocytes overrode the nocodazole-induced MI arrest, whereas reintroduction of USP8 restored MI arrest (Fig. 2F), supporting the conclusion that USP8 loss compromises SAC function.

To further exclude the possibility that the observed phenotype was due to off-target effects of the USP8-targeting siRNA and to solidify the experimental results, we performed experiments using a second siRNA sequence specifically targeting USP8 (USP8-siRNA#2), which yielded consistent results (fig. S2). This suggests that the SAC function is impaired in USP8-deficient oocytes.

### Depletion of USP8 induces meiotic spindle defects and chromosome misalignment

**Fig. 3.**
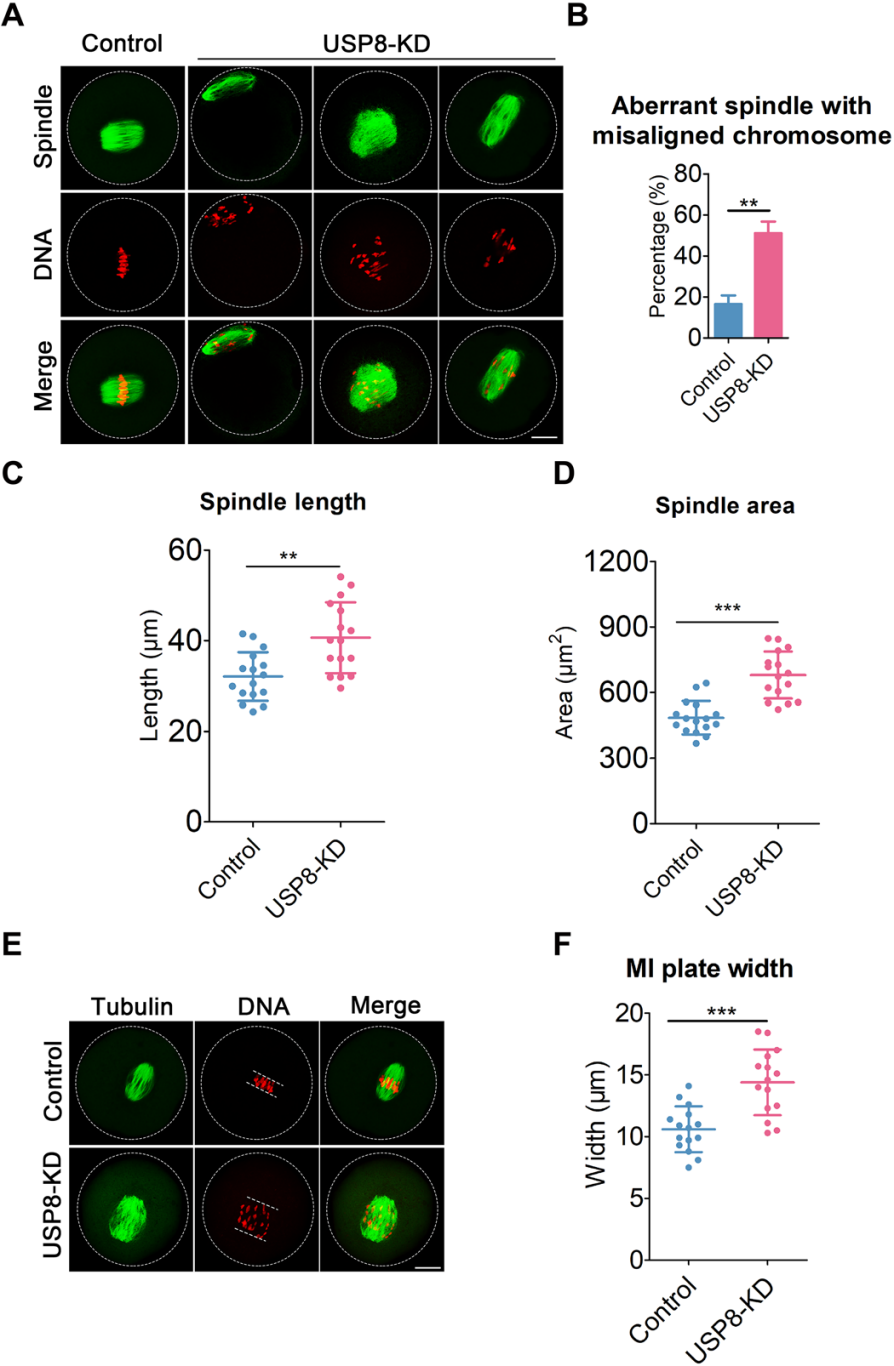
USP8 is indispensable for meiotic spindle assembly and chromosome alignment. (**A**) Representative images of spindle morphologies and chromosome alignment in control and USP8-KD oocytes. At 6 hours post-GVBD, oocytes were fixed and immunostained for α-tubulin and DNA (PI). Scale bar, 20 μm. (**B**) The rates of aberrant spindle with misaligned chromosome were recorded in control (*n* = 49) and USP8-KD (*n* = 52) oocytes. Normal MI oocytes display a characteristic barrel-shaped spindle with properly aligned chromosomes at the equator; if the spindle does not exhibit this typical morphology or if there are “lagging” chromosomes, the oocytes are considered abnormal. (**C** and **D**) The spindle length and area were measured in control (*n* = 16) and USP8-KD (*n* = 16) oocytes at 6 hours post-GVBD. (**E**) Representative images of the width of the MI plate in control and USP8-KD oocytes. At 6 hours after GVBD, oocytes were fixed and immunostained for α-tubulin and DNA (PI). Scale bar, 20 μm. (**F**) The width of the MI plate was measured in control (*n* = 15) and USP8-KD (*n* = 15) oocytes. Data of (B) were presented as the mean percentage (means ± SEM) of at least three independent experiments. Data of [(C), (D), and (F)] were presented as or the mean value (means ± SD) of at least three independent experiments. **P < 0.01; ***P < 0.001.

Considering that the SAC functions as a surveillance mechanism for spindle assembly and chromosome alignment, we subsequently investigated the effect of USP8 deficiency on spindle assembly and chromosome alignment. To achieve this, mouse oocytes from both control and USP8-depleted groups were subjected to immunolabeling with anti–α-tubulin antibody to delineate the spindle morphologies, and were counterstained with propidium iodide (PI) to examine the chromosome alignment. Notably, although control oocytes at the MI stage generally display a characteristic barrel-shaped spindle and properly aligned chromosomes at the equator (Fig. 3A), a marked rise in the incidence of spindle abnormalities and chromosome misalignment was detected in USP8-depleted oocytes (Fig. 3, A and B). The spindle length and area in oocytes at the MI stage were notably enlarged in USP8-depleted oocytes (Fig. 3, C and D). Given that any deviation from normal spindle size reflects an imbalance in spindle dynamics, this defect likely leads to aberrant kinetochore-microtubule (K-MT) attachments. Furthermore, we assessed the width of the chromosome plate at the MI stage and found it to be significantly wider in USP8-depleted oocytes compared to the controls (Fig. 3, E and F).

### USP8 is crucial for maintaining K-MT attachments and euploidy in oocytes

**Fig. 4.**
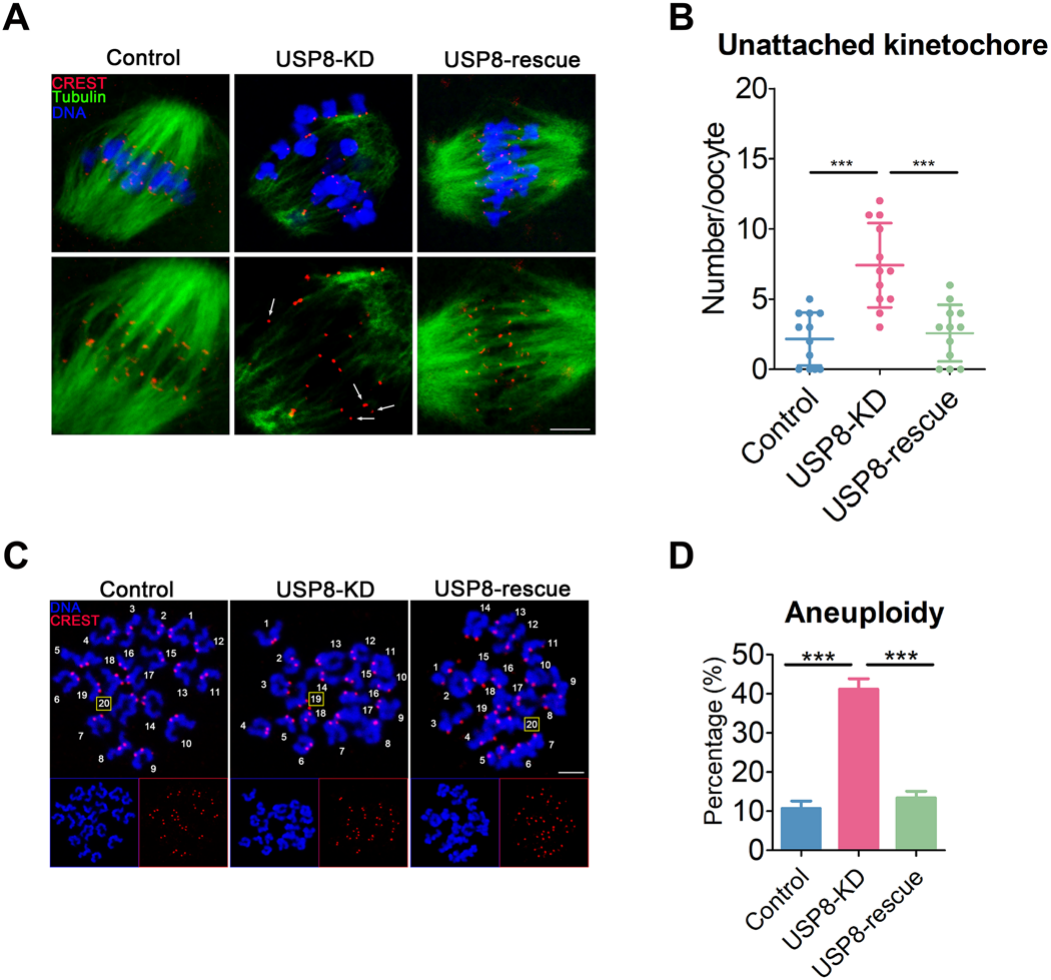
Depletion of USP8 compromises K-M attachments and generates aneuploidy in mouse oocytes. (**A**) Representative images of K-MT attachment in control, USP8-KD, and USP8-rescue oocytes. At 6 hours after GVBD, oocytes were incubated in M2 medium at 4°C for 10 min to induce the depolymerization of unstable microtubules and then immediately fixed and immunostained for α-tubulin, CREST, and DNA (Hoechst). White arrows indicate nonconnected kinetochores. Scale bar, 2.5 μm. (**B**) The number of unattached kinetochores was recorded in control (*n* = 12), USP8-KD (*n* = 12), and USP8-rescue (*n* = 12) oocytes. (**C**) Representative images of euploid and aneuploid MII eggs. Chromosome spreading was performed to count the number of chromosomes in control, USP8-KD, and USP8-rescue oocytes at 10 hours after GVBD. The total number of univalents is indicated by the yellow square. Scale bar, 7 μm. (**D**) The rates of aneuploid eggs were recorded in control (*n* = 30), USP8-KD (*n* = 34), and USP8-rescue (*n* = 29) oocytes. Data of (D) were presented as the mean percentage (means ± SEM) of at least three independent experiments. Data of (B) were presented as the mean value (means ± SD) of at least three independent experiments. ***P < 0.001.

The significantly elevated frequency of chromosome misalignment in USP8-depleted oocytes suggests that K-MT attachments may be compromised. To investigate this, oocytes were subjected to cold treatment to induce depolymerization of microtubules not connected to kinetochores. We observed that, in most control oocytes, microtubules successfully captured the kinetochores on the aligned bivalents at the equator (Fig. 4, A and B). In contrast, USP8-depleted oocytes exhibited a higher incidence of unattached kinetochores and a scarcity of cold-stable microtubules (Fig. 4, A and B). These defects were successfully rescued by USP8 complementation (Fig. 4, A and B), indicating that USP8 is essential for proper K-MT attachments during meiosis.

Abnormalities in the SAC also frequently lead to oocyte aneuploidy (*44*, *45*). To determine whether USP8 depletion induces aneuploidy, we performed karyotype analysis on MII-stage oocytes. Most control oocytes displayed a normal set of univalents and were euploid (Fig. 4C), with no signs of premature sister chromatid separation in the chromosome spreads. This suggests that aneuploidy likely originated from the missegregation of homologous chromosomes during meiosis I. In contrast, USP8-depleted oocytes exhibited a significantly higher incidence of aneuploidy, containing more or fewer than 20 univalents compared to controls (Fig. 4D). This result indicates an essential role for USP8 in preventing aneuploidy. Accordingly, restoring USP8 expression in the knockdown oocytes reduced the aneuploidy rate to a level comparable to that of the controls (Fig. 4D).

### USP8 interacts with BUB3 and is essential for maintaining its protein levels

**Fig. 5.**
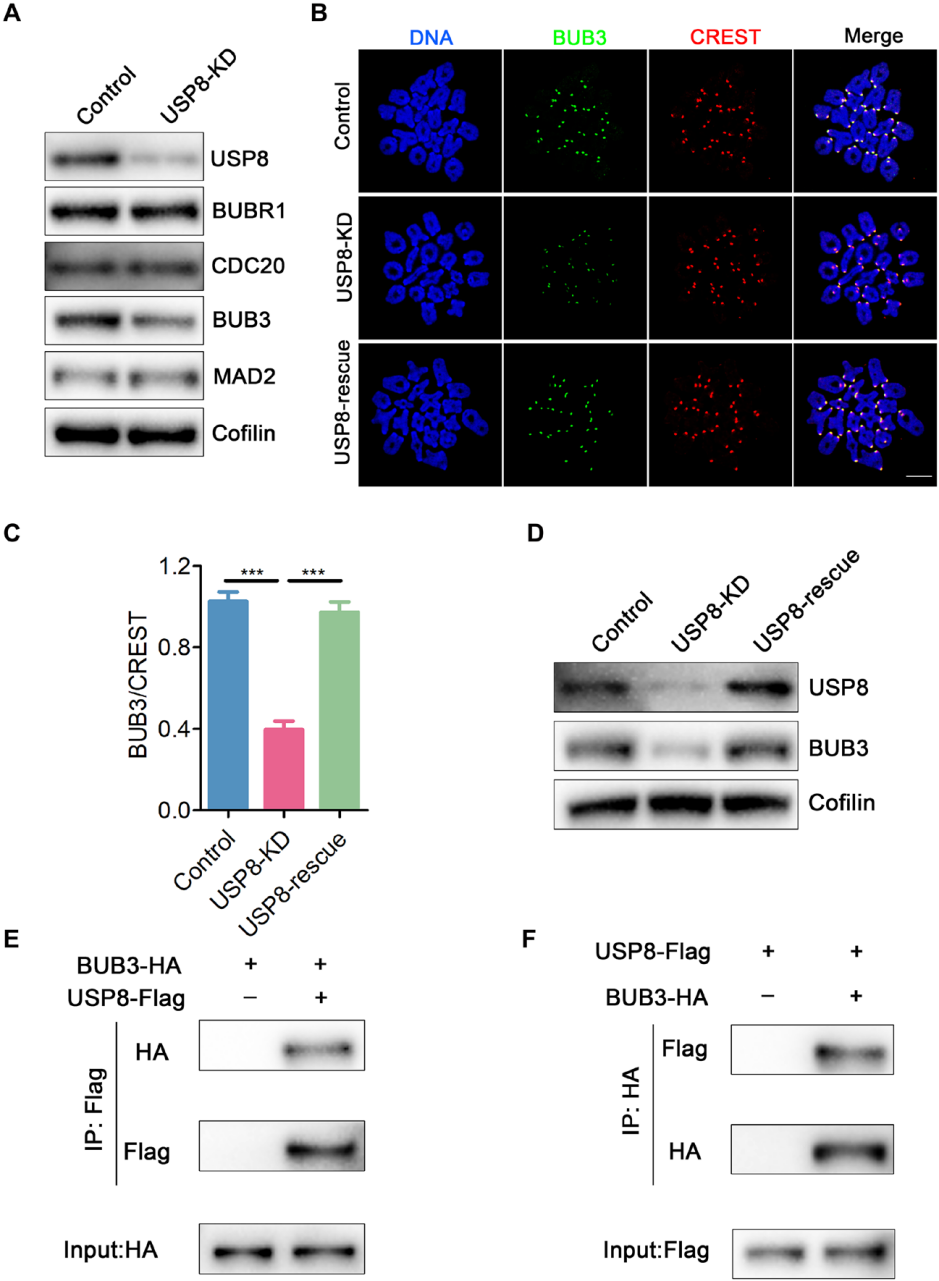
USP8 interacts with BUB3 and is crucial for sustaining BUB3 protein levels. (**A**) Protein levels of the MCC were assessed by immunoblots in control and USP8-depleted oocytes. The blots were probed with USP8, BUBR1, CDC20, BUB3, MAD2, and Cofilin antibodies. (**B**) Localization of BUB3 at the prometaphase I stage in control, USP8-KD, and USP8-rescue oocytes. At 3 hours after GVBD, oocytes were fixed and immunostained for BUB3, CREST, and DNA (Hoechst). Scale bar, 10 μm. (**C**) The relative fluorescence intensities of BUB3 to CREST were measured in control (*n* = 200, kinetochores), USP8-KD (*n* = 200, kinetochores), and USP8-rescue (*n* = 200, kinetochores) oocytes. The signal intensity of BUB3 was normalized with that of CREST. (**D**) Protein levels of BUB3 were assessed by immunoblots in control, USP8-KD, and USP8-rescue oocytes. (**E**) Co-IP result showing the USP8 interaction with BUB3 in oocytes. Mouse oocytes were microinjected with USP8-Flag and BUB3-HA cRNA together or USP8-Flag cRNA alone, maintained for a further 4 hours in 200 μM IBMX to allow time for translation. Target proteins were immunoprecipitated using anti-Flag beads and subjected to Western blotting with Flag and HA antibodies. Input oocyte lysates were immunoblotted with anti-HA antibody to determine the expression of BUB3. (**F**) Co-IP result showing the BUB3 interaction with USP8 in oocytes. Mouse oocytes were microinjected with BUB3-HA and USP8-Flag cRNA together or BUB3-HA cRNA alone, maintained for a further 4 hours in 200 μM IBMX to allow time for translation. Target proteins were immunoprecipitated using anti-HA beads and subjected to Western blotting with HA and Flag antibodies. Input oocyte lysates were immunoblotted with anti-Flag antibody to determine the expression of USP8. Data were presented as the mean percentage (means ± SEM) of at least three independent experiments. ***P < 0.001.

The SAC is a crucial mechanism for maintaining euploidy in oocytes. Given that USP8 deficiency may impair SAC function, we subsequently assessed the protein levels of key SAC proteins BUBR1, BUB3, MAD2, and CDC20, which are components of the mitotic checkpoint complex (MCC) (*52*) in oocytes (*44*, *45*). Immunoblotting revealed that siRNA specifically targeting USP8 effectively knocked down the USP8 protein in oocytes. The depletion of USP8 did not affect the protein levels of BUBR1, MAD2, and CDC20, but the protein level of BUB3 was significantly reduced (Fig. 5A and fig. S3). In addition, we detected the SAC proteins MPS1 and BUB1 in USP8-depleted oocytes, and their protein levels were not affected (fig. S4). We further examined the localization of BUB3 in prometaphase oocytes depleted of USP8. Immunostaining showed that USP8 depletion did not change the localization of BUB3 at kinetochores, but its fluorescence signal was significantly diminished (Fig. 5, B and C, and fig. S5). As expected, reduced levels of BUB3 could be restored by expressing exogenous USP8 in USP8-depleted oocytes, as judged by immunostaining and immunoblots (Fig. 5, B to D). Using similar approaches, we assessed the localization of KNL1, BUBR1, and MAD2. Although KNL1 abundance at kinetochores was unchanged in USP8-depleted oocytes (fig. S6, A and B), the kinetochore levels of both BUBR1 and MAD2 were significantly reduced, despite their total protein levels remaining unaffected (Fig. 5A and fig. S6, C to F). Consistent with SAC deficiency, another line of evidence is the premature degradation of Cyclin B1 in USP8-depleted oocytes (fig. S6, G and H).

The Trim-Away technique enables direct and specific protein degradation (*53*, *54*). To further solidify our conclusions, we used this approach to deplete USP8, which validated our key experimental findings and yielded consistent results with the siRNA-mediated knockdown (fig. S7).

Given the absence of transcriptional activity during oocyte meiotic maturation, we examined whether USP8 interacts with BUB3 to maintain its protein stability. By microinjecting exogenously expressed USP8 and BUB3 into oocytes and performing coimmunoprecipitation (Co-IP) experiments, we demonstrated that USP8 and BUB3 interact in oocytes (Fig. 5E). Reciprocal experiments confirmed their interaction (Fig. 5F). Given that USP8 contains three functional domains (*55*), we sought to identify which of these domains mediates the interaction with BUB3. We divided USP8 into three segments: the N-terminal segment containing the MIT domain, the middle segment containing the RHOD domain along with both the SH3-BM and 14-3-3-BM motifs, and the C-terminal segment containing the catalytic USP domain. Only the N-terminal segment interacted with BUB3, whereas the other two segments did not (fig. S8, A to C), suggesting that the MIT domain of USP8 is responsible for its specific binding to BUB3. Furthermore, endogenous Co-IP confirmed the interaction between USP8 and BUB3 (fig. S8D).

### Augmentation of the BUB3 protein level rescues USP8 depletion-induced meiotic defects and aneuploidy

**Fig. 6.**
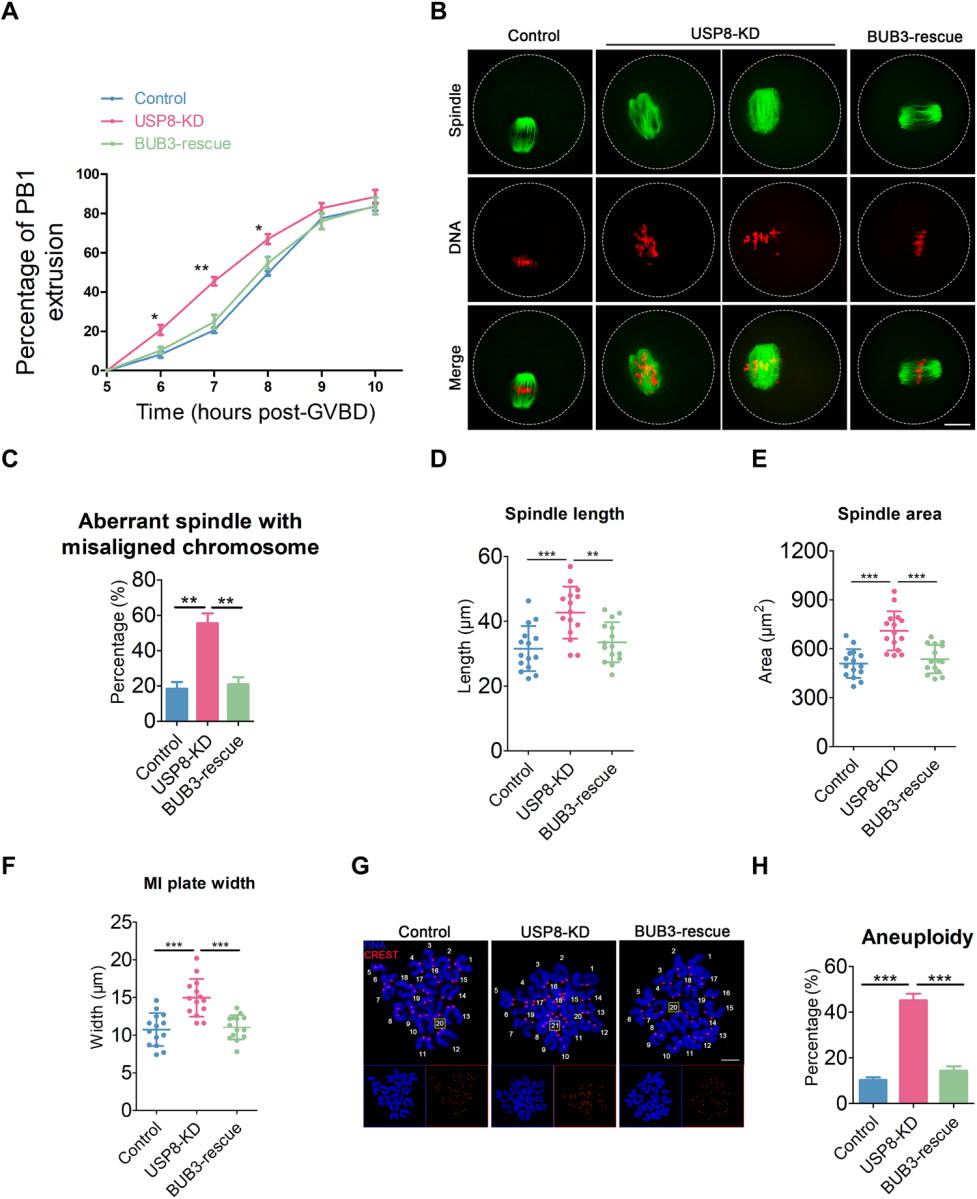
Meiotic defects in USP8-depleted oocytes could be rescued by expression of exogenous BUB3. (**A**) Quantitative analysis of PBE rates were shown in control (*n* = 121), USP8-KD (*n* = 121), and BUB3-rescue (*n* = 117) oocytes at consecutive time points of post-GVBD. (**B**) Representative images of spindle morphologies and chromosome alignment in control, USP8-KD, and BUB3-rescue oocytes. At 6 hours post-GVBD, oocytes were fixed and immunostained for α-tubulin and DNA (PI). Scale bar, 20 μm. (**C**) The rates of aberrant spindle with misaligned chromosome were recorded in control (*n* = 49), USP8-KD (*n* = 50), and BUB3-rescue (*n* = 43) oocytes. (**D** and **E**) The spindle length and area were measured in control (*n* = 15), USP8-KD (*n* = 15), and BUB3-rescue (*n* = 15) oocytes at 6 hours post-GVBD. (**F**) The width of the MI plate was measured in control (*n* = 14), USP8-KD (*n* = 14), and BUB3-rescue (*n* = 14) oocytes. (**G**) Representative images of euploid and aneuploid MII eggs. Chromosome spreading was performed to count the number of chromosomes in control, USP8-KD, and BUB3-rescue oocytes at 10 hours after GVBD. The total number of univalents is indicated by the yellow square. Scale bar, 7 μm. (**H**) The rates of aneuploid eggs were recorded in control (*n* = 30), USP8-KD (*n* = 29), and BUB3-rescue (*n* = 27) oocytes. Data were presented as the mean percentage (means ± SEM) of at least three independent experiments. *P < 0.05; **P < 0.01; ***P < 0.001.

If the decreased BUB3 levels resulting from USP8 depletion led to meiotic defects and aneuploidy, then supplementing USP8-depleted oocytes with exogenous BUB3 should reverse the normal phenotype. We tested this hypothesis by expressing BUB3 in USP8-depleted oocytes (BUB3-rescue oocytes) and monitoring meiotic progression. BUB3 rescue suppressed the premature PBE observed at 6 to 8 hours post-GVBD, restoring the timing to that of control oocytes (Fig. 6A). Successful BUB3 restoration was confirmed by Western blot (fig. S9A). Exogenous BUB3 also counteracted the increased spindle defects and chromosome misalignment, reducing them to control levels (Fig. 6, B and C), and corrected MI defects in spindle length, area, and chromosome plate width (Fig. 6, D to F). Ultimately, this intervention prevented the high aneuploidy incidence caused by USP8 deficiency, bringing it back to control levels (Fig. 6, G and H). We then asked whether the SAC deficiency in aged oocytes, a known aging hallmark (*56*), involves USP8-mediated BUB3 regulation. Western blot analysis, however, showed no significant changes in USP8 or BUB3 protein levels in aged compared to young oocytes (fig. S9B).

In conclusion, the aforementioned experimental results demonstrate that reinstating BUB3 protein levels can rescue the defects resulting from USP8 depletion in oocytes, implying that USP8’s function in regulating SAC in oocytes is mediated by BUB3.

### USP8 stabilizes BUB3 protein levels through deubiquitination in oocytes

**Fig. 7.**
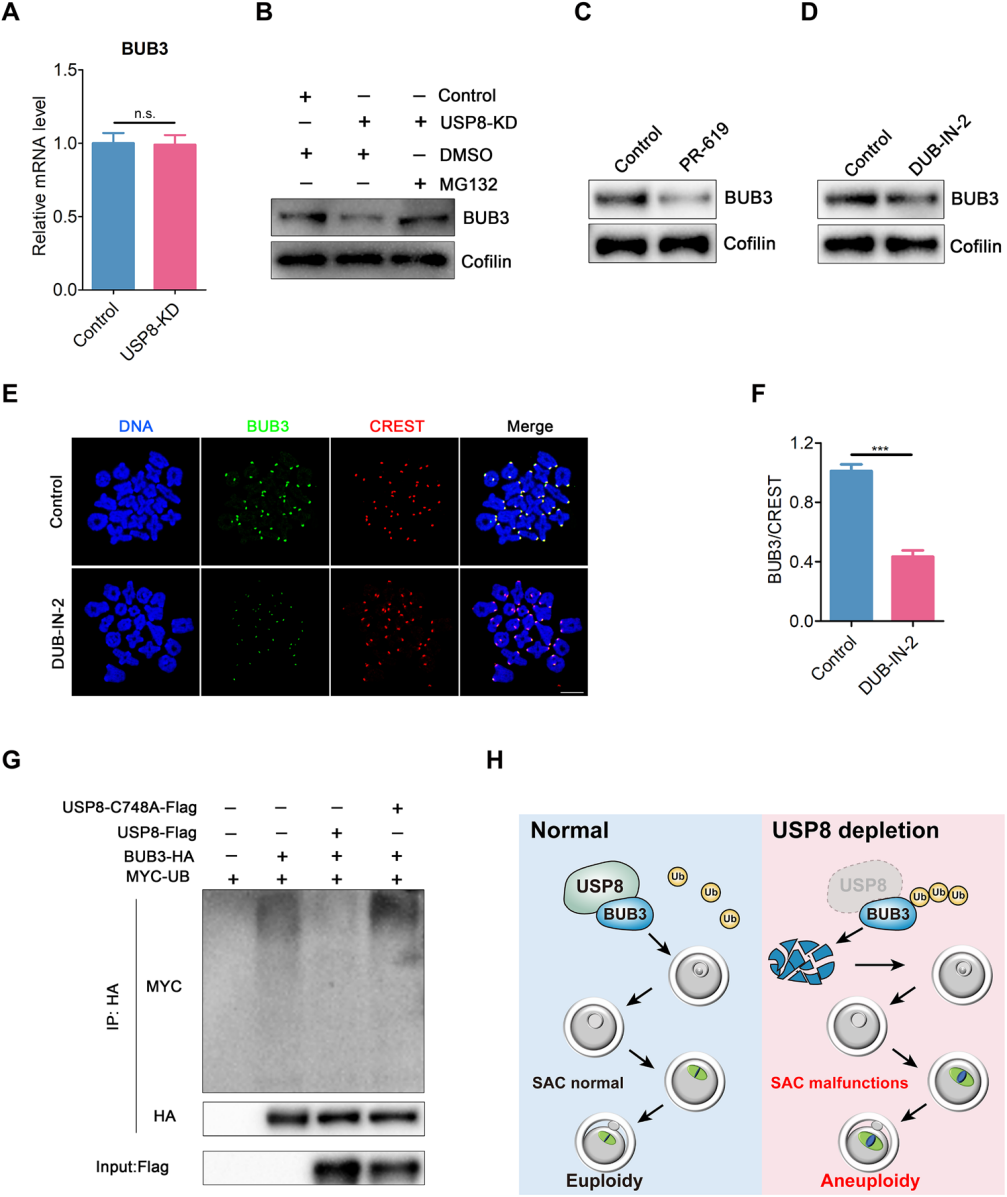
USP8 maintains BUB3 protein levels via deubiquitination in oocytes. (**A**) mRNA levels of BUB3 were determined by reverse transcription quantitative polymerase chain reaction in control and USP8-KD oocytes. (**B**) Protein levels of BUB3 in control, USP8-KD, and USP8-KD + MG132 (25 μM) oocytes. The blots were probed with BUB3 and Cofilin antibodies. DMSO, dimethyl sulfoxide. (**C**) Immunoblotting analysis showing BUB3 protein levels in control oocytes and oocytes treated with the broad-spectrum DUB inhibitor PR-619 (10 μM). (**D**) Immunoblotting analysis of BUB3 protein levels in control and DUB-IN-2 (20 μM)–treated oocytes, where DUB-IN-2 is a specific inhibitor of USP8 deubiquitinating activity. (**E**) Localization of BUB3 at the prometaphase I stage in control and DUB-IN-2–treated oocytes. At 3 hours after GVBD, oocytes were fixed and immunostained for BUB3, CREST, and DNA (Hoechst). Scale bar, 10 μm. (**F**) The relative fluorescence intensities of BUB3 to CREST were measured in control (*n* = 200, kinetochores) and DUB-IN-2–treated (*n* = 200, kinetochores) oocytes. The signal intensity of BUB3 was normalized with that of CREST. ***P < 0.001. (**G**) USP8 reduces the ubiquitination level of BUB3. Mouse oocytes were injected with the respective cRNAs and maintained for an additional 4 hours in 200 μM IBMX to allow for protein translation. Target proteins were immunoprecipitated using anti-HA beads and analyzed by Western blotting with anti-MYC and anti-HA antibodies. Input oocyte lysates were immunoblotted with an anti-Flag antibody to assess USP8 expression. (**H**) Working model of the mechanism that USP8 governs SAC to ensure oocyte euploidy. In normal oocytes, USP8 maintains BUB3 protein levels through its deubiquitinating activity, thereby sustaining the SAC activity in oocytes and ultimately preserving oocyte ploidy. However, in the absence of USP8, BUB3 is targeted for ubiquitin-mediated proteasomal degradation, leading to SAC inactivation and the production of aneuploid eggs.

After USP8 depletion, mRNA levels of BUB3 remained unchanged (Fig. 7A), but ubiquitination and subsequent degradation were perturbed. Application of the proteasome inhibitor MG132 to USP8-depleted oocytes significantly elevated BUB3 levels in immunoblots (Fig. 7B), indicating that USP8 controls BUB3 stability via proteasome-dependent degradation.

USP8 is known to have deubiquitinating activity during mitosis (*19*, *25*). Therefore, we hypothesized that USP8 might maintain the protein stability of BUB3 in oocytes by deubiquitinating it. To verify this hypothesis, we treated oocytes with PR-619, a broad-spectrum deubiquitinase (DUB) inhibitor, and observed a decrease in BUB3 protein levels (Fig. 7C), suggesting that deubiquitinating activity is involved in the protein homeostasis of BUB3 in oocytes. Subsequently, we treated oocytes with DUB-IN-2, a specific inhibitor of USP8 deubiquitinating activity, and found a significant reduction in BUB3 protein levels (Fig. 7D), whereas the levels of USP8 and other components of the MCC remained unchanged (fig. S10A). Moreover, treatment of oocytes with DUB-IN-2 diminished the signal intensity of BUB3 at the kinetochores (Fig. 7, E and F), indicating that USP8 protects BUB3 from ubiquitin-proteasome degradation through its deubiquitination activity in oocytes.

Because the regulation of BUB3 stability by USP8 likely involves USP8’s deubiquitinating activity, we generated a catalytically inactive USP8 mutant (*41*). This mutant failed to rescue the USP8 depletion-induced phenotypes, including the accelerated meiotic progression (fig. S10B), the reduced BUB3 intensity at kinetochores (fig. S10, C and D), and the increased aneuploidy rate (fig. S10, E and F). Furthermore, ubiquitination assays demonstrated that wild-type USP8, but not the catalytically inactive mutant, could reduce the ubiquitination level of BUB3 (Fig. 7G). In summary, our findings indicate that USP8 deubiquitinates and stabilizes BUB3 in oocytes.

## DISCUSSION

Meiosis comprises one round of DNA replication event succeeded by two successive nuclear and cell divisions—meiosis I and meiosis II (*45*, *57*). Chromosome cohesion is established during DNA replication and recombination in fetal oocytes. Postnatally, in meiotic prophase, homologous chromosomes pair and undergo recombination to form a bivalent (*58*, *59*). Meiosis I is unique in that it exclusively separates bivalents, whereas sister chromatids remain associated. Unraveling the mechanisms behind this distinctive chromosome segregation is pivotal for comprehending germ cell development (*60*).

This study reveals USP8 as a previously unreported regulator of the SAC, safeguarding oocyte euploidy via its deubiquitination function. Notably, USP8 depletion did not alter GVBD frequency but induced premature extrusion of the first polar body. Prior investigations established a significant association between premature PBE and defective SAC activity in oocytes (*61*–*64*). In our analyses, we observed that SAC function is compromised and associated with multiple meiotic abnormalities in USP8-deficient oocytes—including aberrant spindle assembly, chromosome misalignment, and incorrect K-MT attachments that result in aneuploidy. These data demonstrate that USP8 is essential for SAC regulation and meiotic progression during oocyte meiosis I.

To further elucidate the role of USP8 in SAC regulation, we examined the protein levels of the MCC components—BUBR1, BUB3, MAD2, and CDC20—in USP8-deficient oocytes. The protein levels of BUBR1, MAD2, and CDC20 remained unchanged. Unexpectedly, BUB3 levels were reduced, although its localization at kinetochores was unaffected. Given the absence of transcriptional activity during oocyte meiotic maturation, we explored whether USP8 interacts with BUB3 to stabilize its protein levels. Co-IP experiments revealed that USP8 interacts with BUB3 in oocytes. Moreover, reintroducing exogenous BUB3 rescued the meiotic defects caused by USP8 depletion. These results indicate that USP8’s role in SAC control is mediated through BUB3.

Last, we demonstrate that USP8 stabilizes BUB3 protein levels through deubiquitination in oocytes. Blocking the ubiquitin-proteasome degradation pathway increased protein levels of BUB3 in USP8-depleted oocytes. Inhibition of deubiquitination activity in oocytes reduces the protein level of BUB3, implying that deubiquitination activity is involved in the protein homeostasis of BUB3. Furthermore, specific inhibition of the deubiquitination activity of USP8 also leads to a decrease in the protein level of BUB3 and its fluorescence intensity at the kinetochore in oocytes. These findings are interesting because, until now, it was only clearly established that the SAC closely cooperates with ubiquitination activity to maintain the euploidy of oocytes (*44*, *45*). However, whether deubiquitination participates in SAC regulation remains unknown. Our study provides direct evidence to address this scientific question. Compared with somatic cells, oocytes have many unique characteristics, such as their large size, longer division phase, and unique mechanisms of chromosome segregation and cytokinesis (*44*, *45*, *62*). A thorough investigation into the coordinated regulation of ubiquitination activity, deubiquitination activity, and the SAC will be an important research topic. It will provide theoretical support for the mechanisms underlying the production of aneuploid oocytes in humans and the resulting trisomy syndromes and miscarriages. Furthermore, as oocyte quality depends not only on the regulation of nuclear maturation but also critically on cytoplasmic maturation, whether and how deubiquitination participates in the regulatory mechanisms of cytoplasmic maturation represents an important area for future investigation.

In summary, we uncover a previously unknown function of the deubiquitinase USP8 in preserving oocyte euploidy by regulating the SAC. Specifically, we demonstrate that USP8 stabilizes its newly identified substrate BUB3 through deubiquitination (Fig. 7H), revealing an essential contribution of deubiquitination to SAC activity in preventing aneuploid egg formation.

## MATERIALS AND METHODS

### Animals

Six- to 8-week-old female ICR mice were used in all experiments (except where explicitly stated as aged mice, which were 10-month-old female ICR mice). All animal experiments were approved by the Animal Care and Use Committee of Guangdong Second Provincial General Hospital (approval no. 2022-DW-KZ-068 to C.Z.) and performed in accordance with institutional guidelines.

### Oocyte collection and culture

As previously described (*51*, *59*), female ICR mice were euthanized by cervical dislocation. Fully grown oocytes arrested at prophase of meiosis I were collected from ovaries in M2 medium (Sigma-Aldrich, St. Louis, MO, USA). Only those immature oocytes displaying a GV were further cultured in M16 medium (Sigma-Aldrich) under liquid mineral oil at 37°C in an atmosphere of 5% CO_2_. At different time points after culture, oocytes were collected for subsequent analysis.

### siRNA knockdown

USP8-targeting siRNA antisense oligo (Genepharma, Shanghai, China; 5′-CCAAGUUUCUUGAUCCAAUTT-3′; USP8-siRNA#2: 5′-GCAGGAUUAUUAUCUUUCATT-3′) was diluted with water to give a working concentration of 25 μM, and then ~5 to 10 pl of oligo was microinjected into the cytoplasm of fully grown GV oocytes using a Narishige microinjector (Tokyo, Japan). A nontargeting siRNA oligo (antisense sequence: 5′-ACGUGACACGUUCGGAGAATT-3′) was injected as a control. To facilitate the degradation of mRNA by siRNA, oocytes were arrested at the GV stage in M16 medium containing 50 μM 3-isobutyl-1-methylxanthine (IBMX) for 20 hours and then transferred to IBMX-free M16 medium to resume the meiosis for subsequent experiments.

### cRNA construct and in vitro transcription

As previously described (*51*, *59*), cDNA was subcloned into pcDNA3.1. Mutant USP8 with three silent third-codon point mutations in the sequence targeted by the seed region of siRNA antisense strand provided an siRNA-resistant construct. Capped complementary RNA (cRNA) was synthesized from linearized plasmid using the T7 mMessage mMachine kit (Thermo Fisher Scientific, Waltham, MA, USA) and purified with MEGAclear kit (Thermo Fisher Scientific). Typically, 10 to 12 pl of cRNA (0.5 to 1.0 μg/μl) was injected into oocytes and then arrested at the GV stage in M16 medium containing 200 μM IBMX for 2 to 4 hours to allow translation before transfer into IBMX-free M16 medium for subsequent studies.

### Immunofluorescence and confocal microscopy

As previously described (*51*, *59*), oocytes were fixed in 4% paraformaldehyde in phosphate-buffered saline (PBS) (pH 7.4) for 30 min and permeabilized in 0.5% Triton X-100 for 20 min at room temperature. Then, oocytes were blocked with 1% bovine serum albumin (BSA)–supplemented PBS for 1 hour and incubated with USP8 (1:100; Abclonal, A7031), BUB3 (1:100; Abcam, ab133699), BUBR1 (1:100; Abcam, Ab28193), MAD2 (1:100; Proteintech, 10337-1-AP), KNL1 (1:100; Abcam, Ab70537), α-tubulin–FITC (1:300; Sigma-Aldrich, F2168), or CREST (1:200; Antibodies Incorporated, CA95617) antibodies at 4°C overnight. After washing in PBST, oocytes were incubated with an appropriate secondary antibody for 1 hour at room temperature. Then, oocytes were counterstained with PI or Hoechst for 10 min. Last, oocytes were mounted on glass slides and observed under a confocal microscope (LSM 900 META, Zeiss, Germany).

For measurement of fluorescence intensity, the signals from both control and treatment oocytes were acquired by performing the same immunostaining procedure and setting up the same parameters of confocal microscope. The average fluorescence intensity per unit area within the region of interest (ROI) was applied to quantify the fluorescence of each oocyte images. Fluorescence intensity was randomly measured by plot profiling using ImageJ software (NIH, USA). Fluorescence intensity on kinetochores was quantified by drawing a circle closely the dot-like CREST staining covering the interested SAC protein staining. The intensity of SAC proteins was normalized against the CREST fluorescence intensity.

### Immunoprecipitation and immunoblotting analysis

As previously described (*51*, *59*), for exogenous Co-IP, 300 mouse oocytes were microinjected with the corresponding cRNA, maintained for a further 4 hours in 200 μM IBMX to allow time for translation. Then, those oocytes were harvested in lysis buffer containing a protease inhibitor cocktail (Invitrogen). Target proteins were immunoprecipitated using anti-Flag or anti-HA beads were incubated together for 16 hours at 4°C. After five washes with wash buffer, the bead-antibody-antigen complex were then resuspended in elution buffer. Samples were supplemented with 4× LDS sample buffer (Thermo Fisher Scientific) and heated at 95°C for 5 min. For endogenous Co-IP, 1800 oocytes were collected in lysis buffer containing a protease inhibitor cocktail (Invitrogen). Target proteins were immunoprecipitated by incubating lysates with anti-USP8 antibody or normal rabbit immunoglobulin G (IgG) as a control on a rotator overnight at 4°C. Protein G magnetic beads were then added and incubated for 4 hours at 4°C with continuous rotation. After five washes with wash buffer, the bead-antibody-antigen complexes were resuspended in elution buffer. The samples were mixed with 4× LDS sample buffer (Thermo Fisher Scientific) and heated at 95°C for 5 min. For immunoblots, samples were separated on 10% Bis-Tris precast gels and transferred onto polyvinylidene difluoride (PVDF) membranes. The blots were further blocked in TBST containing 5% low-fat dry milk for 1 hour at room temperature and then incubated with USP8 (1:500; Abclonal, A7031), USP8 (1:500; Proteintech, 67321-1-Ig), Cyclin B1 (1:1000; Cell Signaling Technology, 4135), BUB3 (1:1000; Abcam, ab133699), BUBR1 (1:1000; Abcam, Ab28193), MAD2 (1:1000; Proteintech, 10337-1-AP), CDC20 (1:500; Proteintech, 10252-1-AP), BUB1 (1:1000; Abcam, Ab195268), MPS1 (1:500; Proteintech, 10381-1-AP), Flag (1:1000; Sigma-Aldrich, F3165), HA (1:1000; Sigma-Aldrich, H9658), MYC (1:1000; Cell Signaling Technology, 2278), Normal Rabbit IgG (1:1000; Cell Signaling Technology, 2729), or Cofilin (1:5000; Proteintech, 66057-1-Ig) antibodies at 4°C overnight. After washing in TBST, the blots were incubated with horseradish peroxidase (HRP)–conjugated secondary antibodies for 1 hour at room temperature. Chemiluminescence was detected with ECL Plus (GE, Piscataway, NJ, USA) and protein bands were acquired by the Tanon-3900 Chemiluminescence Imaging System (Tanon, Beijing, China). Band intensities were quantified using ImageJ software and normalized to loading controls.

### Trim-Away

The Trim-Away technique enables rapid and specific protein depletion, offering a key advantage in circumventing the genetic compensation effects often associated with gene-edited mouse models. To acutely deplete USP8, we adopted a previously reported approach (*53*, *54*). Oocytes were coinjected with in vitro–transcribed Trim21 mRNA (1 μg/μl) and an anti-USP8 antibody (0.85 μg/μl) and then incubated in medium containing 200 μM IBMX for 4 hours to allow for protein degradation.

### Reverse transcription quantitative polymerase chain reaction

As previously described (*51*), 10 to 20 oocytes collected were lysed in cell lysis buffer containing 0.2% Triton X-100 and RNase inhibitor. The lysate was then reverse transcribed using the SuperScript III First-Strand System (Invitrogen, 18080051) following the manufacturer’s instructions. Quantitative polymerase chain reaction (PCR) was conducted with the One Step TBGreen PrimeScript RT-PCR Kit (Takara, RR066A) on the ABI QuantStudio5 Real-Time PCR system (Applied Biosystems). Murine Atcb was applied as an endogenous control. Data were obtained from at least three replicated biological experiments per genotype with three technical repeats each time and were expressed as enrichment of 2^−ΔΔCt^.

### Chromosome spreading

As previously described (*51*), oocytes were exposed to Tyrode’s buffer (pH 2.5) for about 30 s at 37°C to remove zona pellucidae. After recovery in M2 medium for 10 min, oocytes were fixed in a drop of 1% paraformaldehyde with 0.15% Triton X-100 on a glass slide. After air drying, chromosomes were counterstained with Hoechst and examined under a laser scanning confocal microscope.

### Statistical analysis

As previously described (*51*, *59*), all percentages or values from at least three biological replicates were expressed as means ± SEM or means ± SD, and the number of oocytes was labeled in parentheses as (*n*). Data were analyzed by paired-samples *t* test, which was provided by GraphPad Prism 5 statistical software. The level of significance was accepted as *P* < 0.05.
