# Supplementary material for: Deubiquitinase USP8 regulates the spindle assembly checkpoint in oocytes
Source: Sci Adv. 2026 Mar 13;12(11):eaeb2345. doi: 10.1126/sciadv.aeb2345 (PMC12985659; doi:10.1126/sciadv.aeb2345)
Supplement: Supplementary file 1 — Figs. S1 to S10 [file sciadv.aeb2345_sm.pdf]

Science Advances
AAAS

Supplementary Materials for

Deubiquitinase USP8 regulates the spindle assembly checkpoint in oocytes

Changyin Zhou *et al.*

Corresponding author: Changyin Zhou, zhoucy@gd2h.org.cn; Qing-Yuan Sun, sunqy@gd2h.org.cn; Xiang-Hong Ou, ouxh@gd2h.org.cn

Sci. Adv. **12**, eaeb2345 (2026)
DOI: 10.1126/sciadv.aeb2345

This PDF file includes:

Figs. S1 to S10

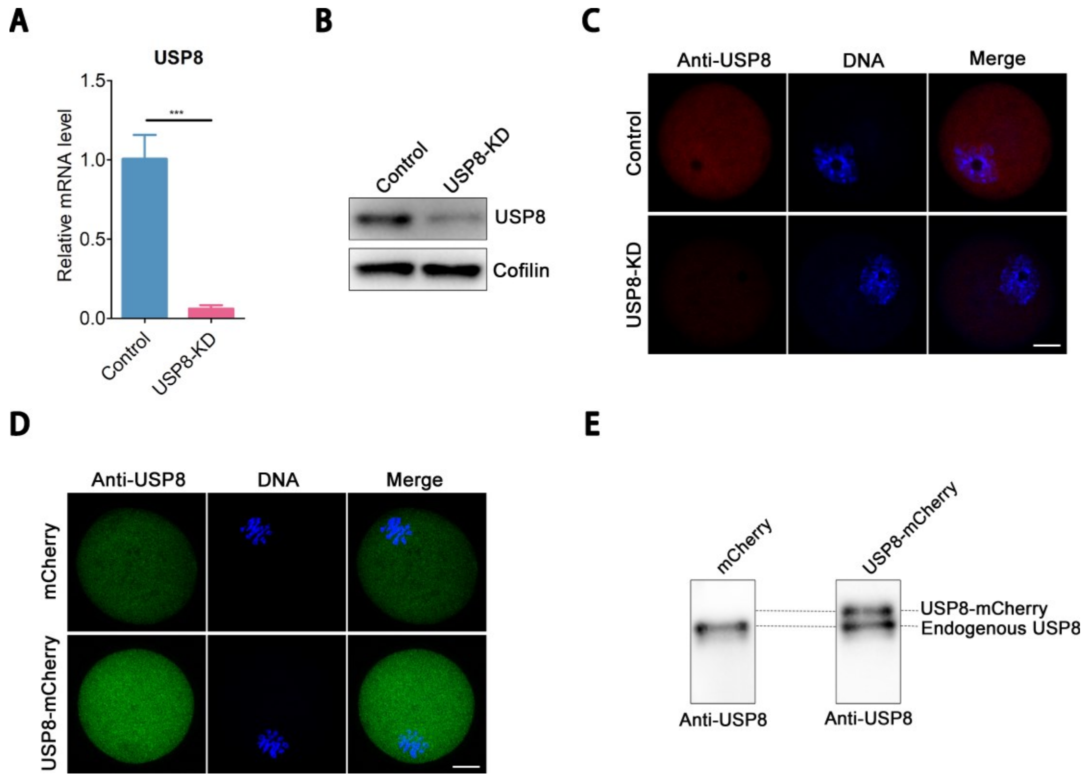

fig. S1. Effects of USP8 knockdown or overexpression in mouse oocytes. (A) Reverse transcription polymerase chain reaction (RT-PCR) showing the Relative mRNA level of USP8 in control and USP8-KD groups. (B) Oocytes in control and USP8-KD groups were collected and immunoblotted for USP8 and Cofilin. (C) Representative images of the localization of endogenous USP8 in control and USP8-KD oocytes. Mouse oocytes were immunostained with USP8 antibody. Scale bar, 20 μm. (D) Representative images showing the localization of USPP8-mCherry during mouse oocyte meiosis. Oocytes were microinjected with either mCherry or USP8-mCherry mRNA at the GV stage, maintained in 50 μM IBMX for 2 hours, and then transferred to IBMX-free medium to allow maturation to the GVBD stage. The oocytes were then immunostained with a primary USP8 antibody, followed by incubation with a green fluorescent secondary antibody. Scale bar, 20 μm. (E) Oocytes were injected with mCherry or USP8-mCherry mRNA and analyzed by Western blot using an anti-USP8 antibody. Data were presented as mean percentage (mean ± SEM) of at least three independent biological replicates. $***P < 0.001$.

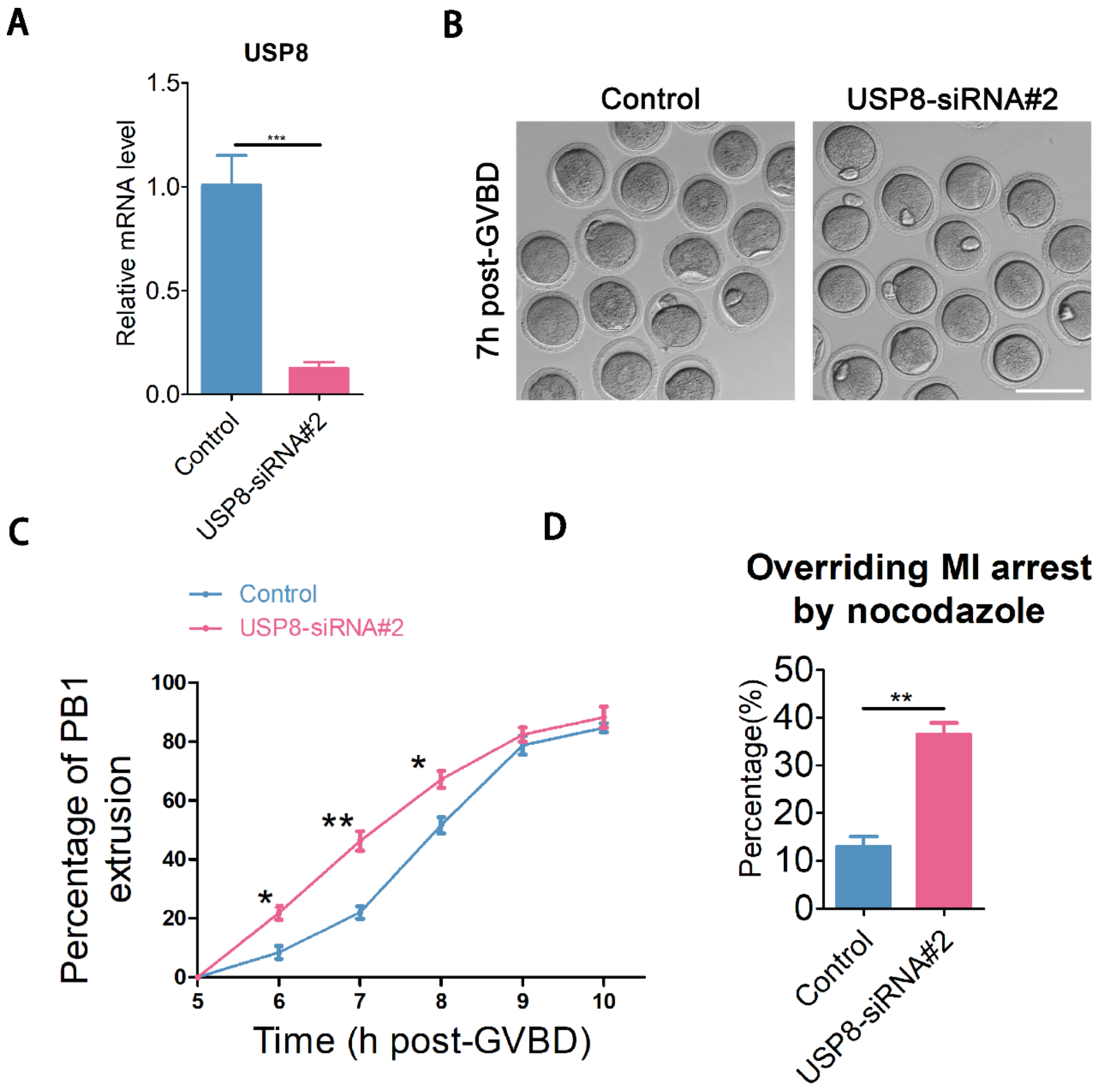

fig. S2. Effect of USP8-siRNA#2 on meiotic progression in mouse oocytes. (A) RT-PCR showing the Relative mRNA level of USP8 in control and USP8-siRNA#2 groups. (B) Representative images of PBE in control and USP8-siRNA#2 oocytes at the time point of 7 h post-GVBD. Scale bar, 100 μm. (C) Quantitative analysis of PBE rates was shown in control (n = 118) and USP8-siRNA#2 (n = 119) oocytes at consecutive time points of post-GVBD. (D) The proportion of oocytes overriding metaphase I arrest following nocodazole treatment was recorded in control (n = 93) and USP8-siRNA#2 (n =93) oocytes. Data were presented as mean value (mean ± SEM) of at least three independent experiments. *$P < 0.05$, **$P < 0.01$, ***$P < 0.001$.

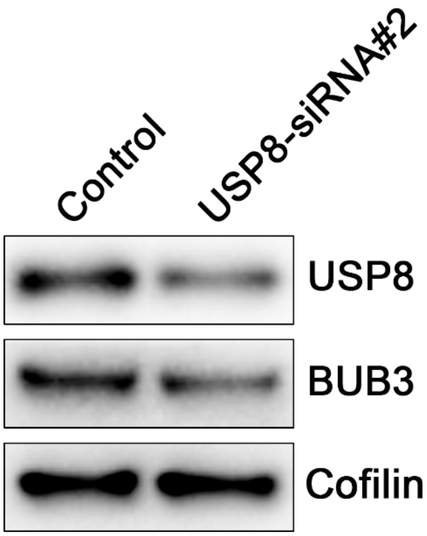

fig. S3. Effect of USP8-siRNA#2 on the expression of BUB3. Protein levels of BUB3 were assessed by immunoblots in control and USP8-siRNA#2 oocytes. The blots were probed with USP8, BUB3, and Cofilin antibodies.

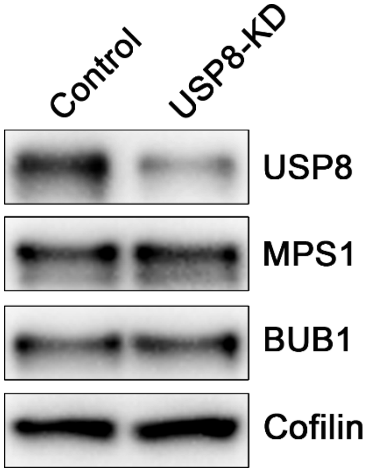

fig. S4. Effect of USP8 depletion on the expression of SAC proteins. Protein levels of MPS1 and BUB1 were assessed by immunoblots in control and USP8-KD oocytes. The blots were probed with USP8, MPS1, BUB1 and Cofilin antibodies.

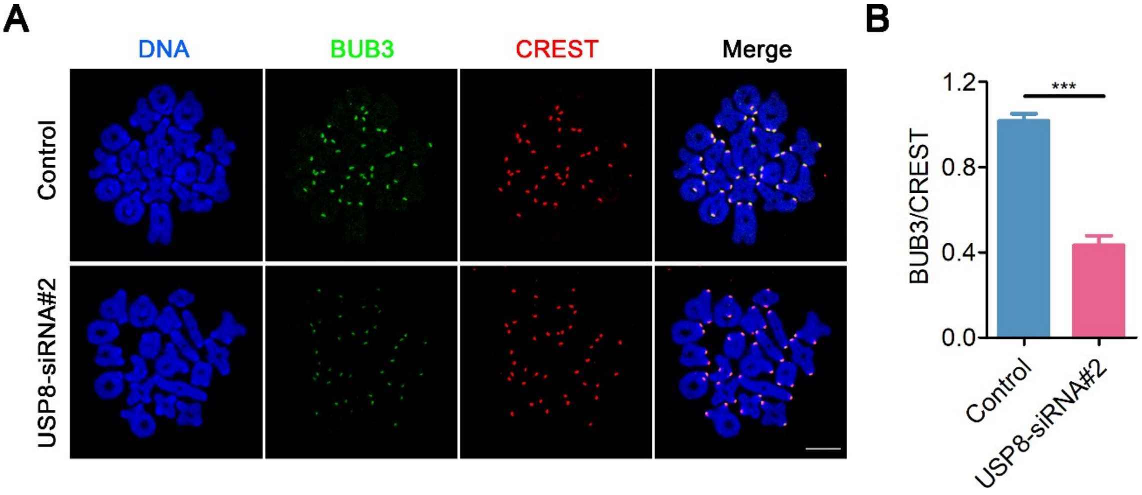

fig. S5. Effect of USP8-siRNA#2 on the localization of BUB3. (A) Localization of BUB3 at prometaphase I stage in control and USP8-siRNA#2 oocytes. At 3 hours after GVBD, oocytes were fixed and immunostained for BUB3, CREST and DNA (Hoechst). Scale bar, 10 μm. (B) The relative fluorescence intensities of BUB3 to CREST were measured in control (n = 160, kinetochores) and USP8-siRNA#2 (n = 160, kinetochores) oocytes. The signal intensity of BUB3 was normalized with that of CREST.

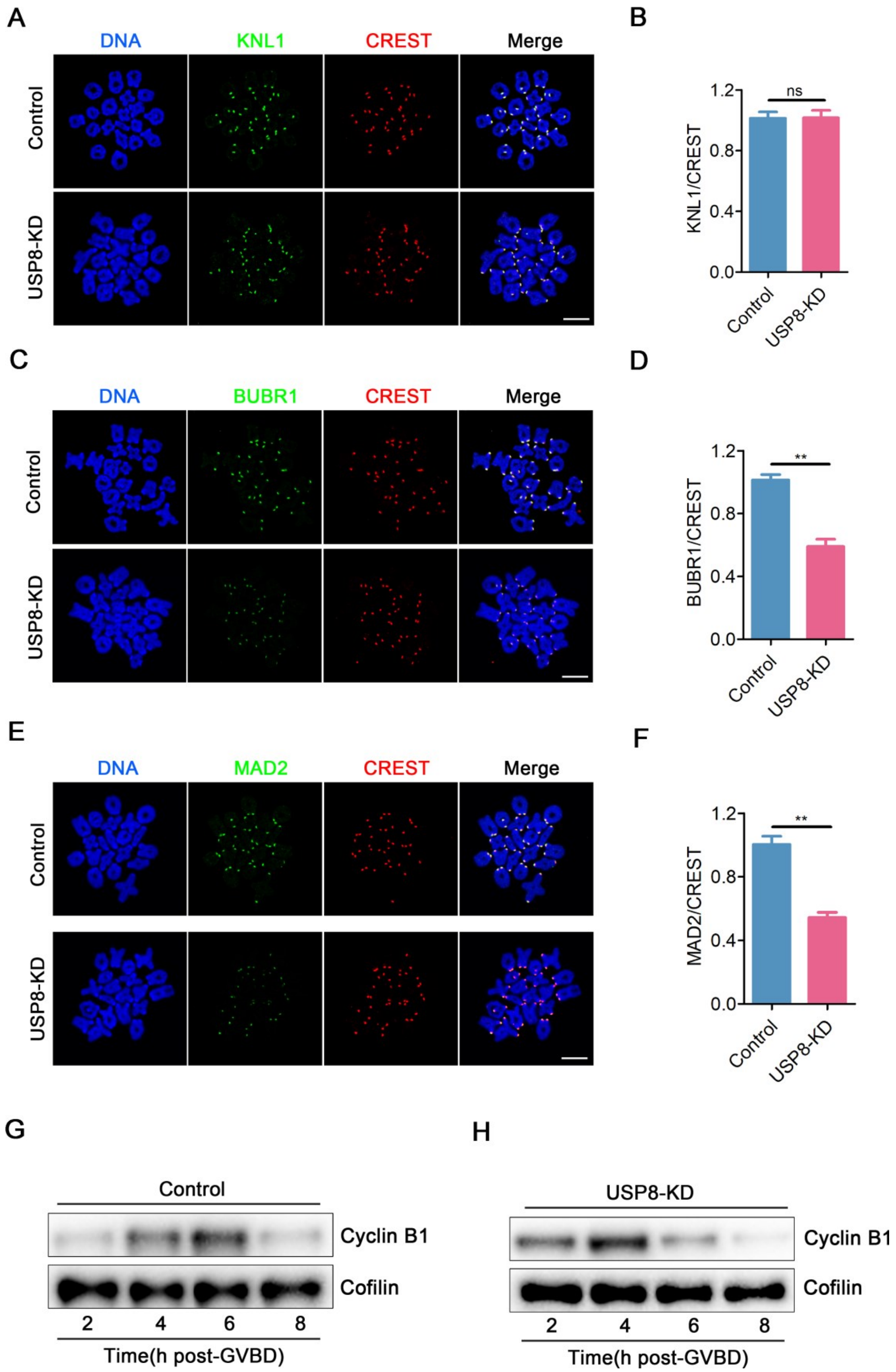
A
DNA
KNL1
CREST
Merge
Control
USP8-KD
B
KNL1/CREST
ns
Control
USP8-KD
C
DNA
BUBR1
CREST
Merge
Control
USP8-KD
D
BUBR1/CREST
**
Control
USP8-KD
E
DNA
MAD2
CREST
Merge
Control
USP8-KD
F
MAD2/CREST
**
Control
USP8-KD
G
Control
Cyclin B1
Cofilin
2 4 6 8
Time(h post-GVBD)
H
USP8-KD
Cyclin B1
Cofilin
2 4 6 8
Time(h post-GVBD)

fig. S6. Effect of USP8 depletion on the localization of SAC proteins and the protein abundance of Cyclin B1. (A, C, and E) Representative images of the localization of KNL1, BUBR1, and MAD2 in control and USP8-KD oocytes, respectively. Scale bar, 10 μm. (B, D, and F) The relative fluorescence intensities of KNL1, BUBR1, and MAD2 compared to CREST were measured in control (n = 200, kinetochores) and USP8-depleted (n = 200, kinetochores) oocytes, respectively. The signal intensity was normalized to CREST. (G and H) The change of Cyclin B1 protein levels was shown in control and USP8-depleted oocytes at different time points of post-GVBD. The blots were probed with Cyclin B1 and Cofilin antibodies. $^{**}P < 0.01$, ns, not significant.

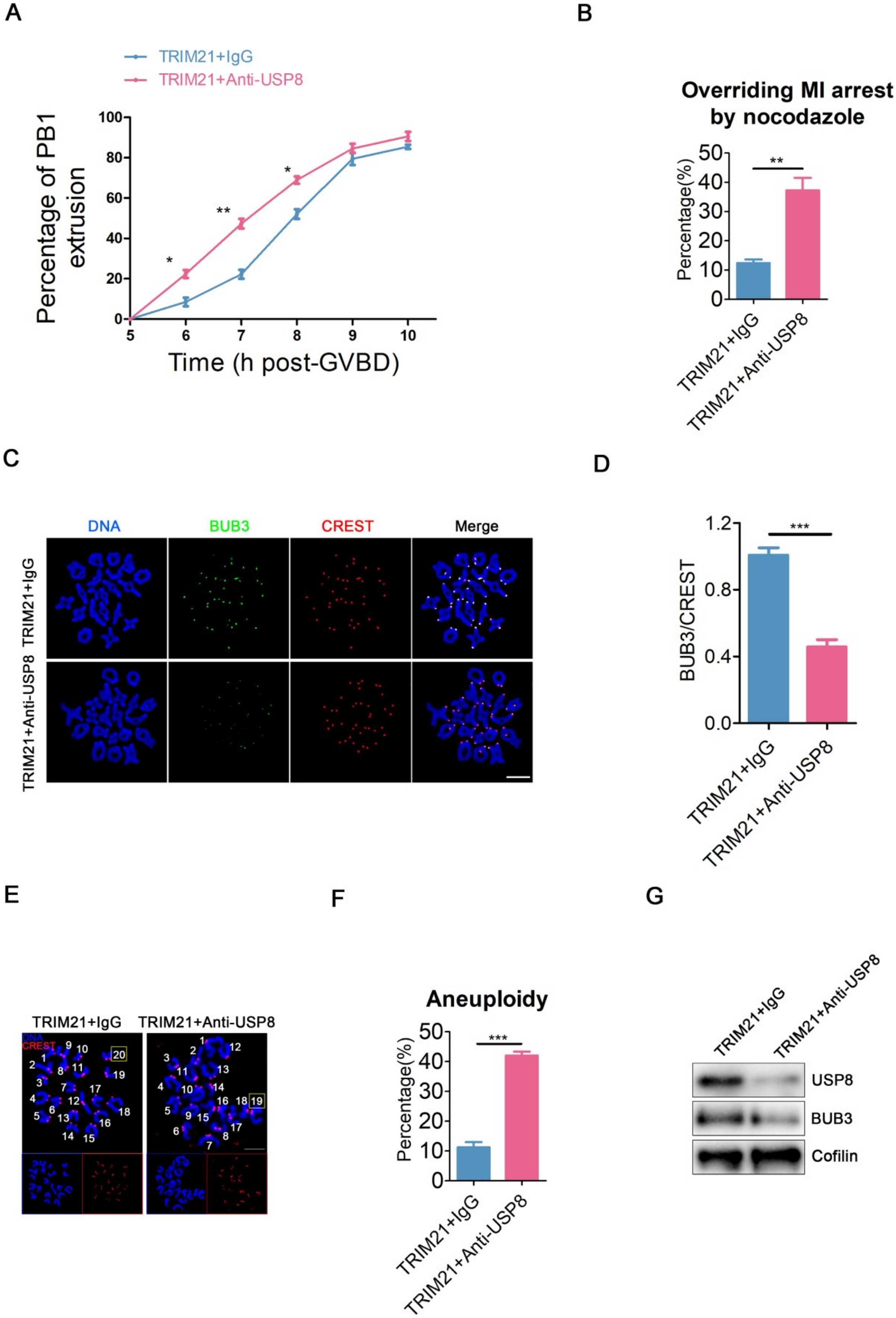
A
TRIM21+IgG
TRIM21+Anti-USP8
Percentage of PB1 extrusion
100
80
60
40
20
0
*
**
*
5
6
7
8
9
10
Time (h post-GVBD)
B
Overriding MI arrest by nocodazole
Percentage(%)
50
40
30
20
10
0
**
TRIM21+IgG
TRIM21+Anti-USP8
C
DNA
BUB3
CREST
Merge
TRIM21+IgG
TRIM21+Anti-USP8
D
BUB3/CREST
1.2
0.8
0.4
0.0
***
TRIM21+IgG
TRIM21+Anti-USP8
E
TRIM21+IgG
TRIM21+Anti-USP8
DNA
CREST
F
Aneuploidy
Percentage(%)
50
40
30
20
10
0
***
TRIM21+IgG
TRIM21+Anti-USP8
G
TRIM21+IgG
TRIM21+Anti-USP8
USP8
BUB3
Cofilin

fig. S7. Effect on oocytes following specific USP8 degradation employing the Trim-Away technique. (A) Quantitative analysis of PBE rates was shown in TRIM21+IgG (n = 117) and TRIM21+Anti-USP8 (n = 116) oocytes at consecutive time points of post-GVBD. (B) The proportion of oocytes overriding metaphase I arrest following nocodazole treatment was recorded in TRIM21+IgG (n = 89) and TRIM21+Anti-USP8 (n = 89) oocytes. (C) Localization of BUB3 at prometaphase I stage in TRIM21+IgG and TRIM21+Anti-USP8 oocytes. At 3 hours after GVBD, oocytes were fixed and immunostained for BUB3, CREST and DNA (Hoechst). Scale bar, 10 μm. (D) The relative fluorescence intensities of BUB3 to CREST were measured in TRIM21+IgG (n = 180, kinetochores) and TRIM21+Anti-USP8 (n = 180, kinetochores) oocytes. The signal intensity of BUB3 was normalized with that of CREST. (E) Representative images of euploid and aneuploid MII eggs. Chromosome spreading was performed to count the number of chromosomes in TRIM21+IgG and TRIM21+Anti-USP8 oocytes at 10 hours after GVBD. The total number of univalents is indicated by the yellow square. Scale bar, 7 μm. (F) The rates of aneuploid eggs were recorded in TRIM21+IgG (n = 28) and TRIM21+Anti-USP8 (n = 31) oocytes. (G) Protein levels of BUB3 were assessed by immunoblots in TRIM21+IgG and TRIM21+Anti-USP8 oocytes. The blots were probed with USP8, BUB3 and Cofilin antibodies. Data were presented as mean percentage (mean ± SEM) of at least three independent experiments. $*P < 0.05$, $**P < 0.01$, $***P < 0.001$.

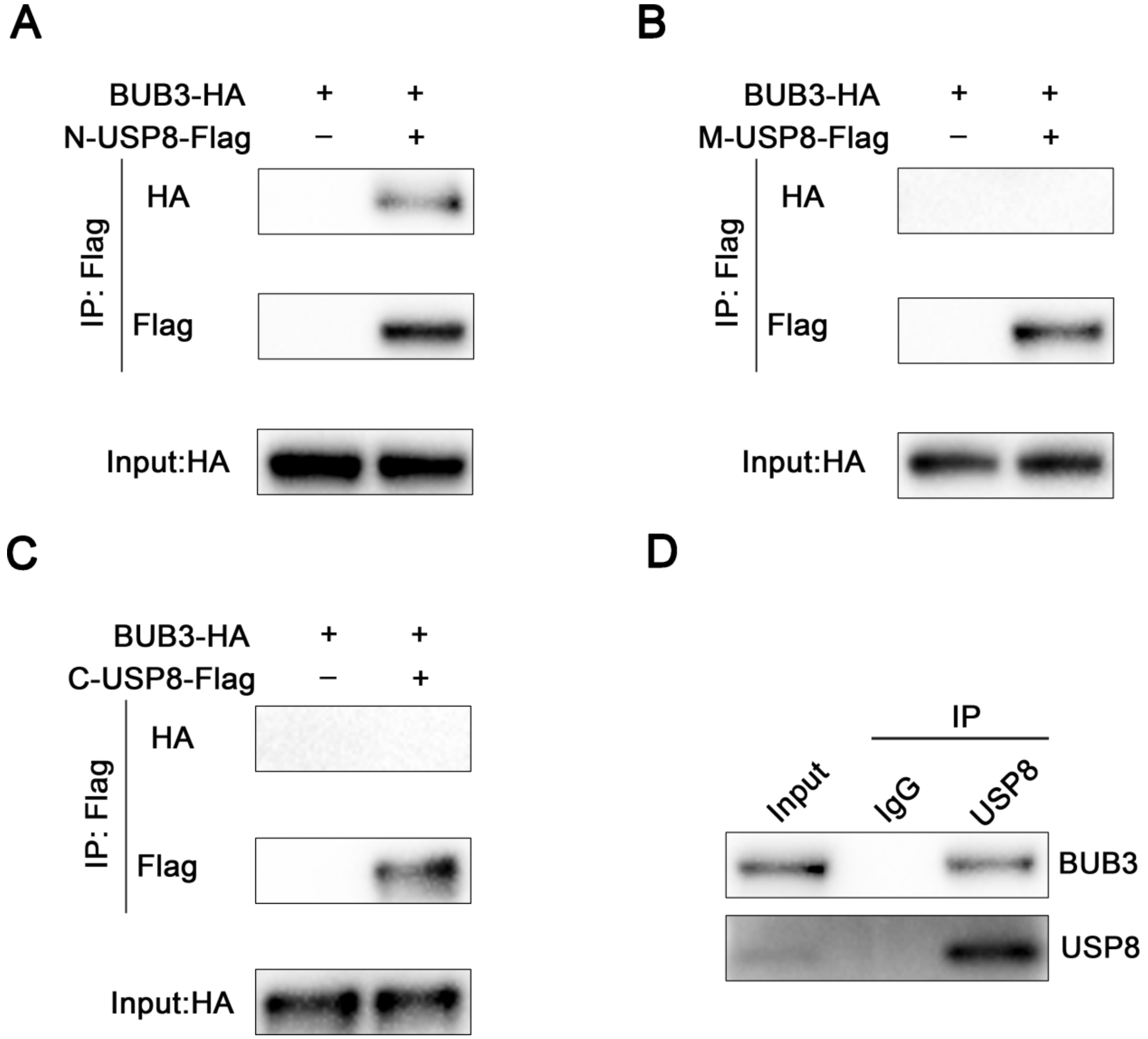

fig. S8. Co-immunoprecipitation result showing USP8 interaction with BUB3 in oocytes. (A) Mouse oocytes were microinjected with N-USP8-Flag (encoding the murine N-terminal fragment of USP8, corresponding to residues 1-120) and BUB3-HA cRNA together or BUB3-HA cRNA alone, maintained for a further 4 hours in 200μM IBMX to allow time for translation. Target proteins were immunoprecipitated using anti-Flag beads and subjected to western blotting with Flag and HA antibodies. Input oocyte lysates were immunoblotted with anti-HA antibody to determine the expression of BUB3. (B) Mouse oocytes were microinjected with M-USP8-Flag

(encoding the murine middle fragment of USP8, corresponding to residues 121-729) and BUB3-HA cRNA together or BUB3-HA cRNA alone, maintained for a further 4 hours in 200μM IBMX to allow time for translation. Target proteins were immunoprecipitated using anti-Flag beads and subjected to western blotting with Flag and HA antibodies. Input oocyte lysates were immunoblotted with anti-HA antibody to determine the expression of BUB3. (C) Mouse oocytes were microinjected with C-USP8-Flag (encoding the murine C-terminal fragment of USP8, corresponding to residues 730-1080) and BUB3-HA cRNA together or BUB3-HA cRNA alone, maintained for a further 4 hours in 200μM IBMX to allow time for translation. Target proteins were immunoprecipitated using anti-Flag beads and subjected to western blotting with Flag and HA antibodies. Input oocyte lysates were immunoblotted with anti-HA antibody to determine the expression of BUB3. (D) Co-IP was performed with IgG and anti-USP8 antibody, respectively. The blots were probed with anti-BUB3 and anti-USP8 antibodies, respectively.

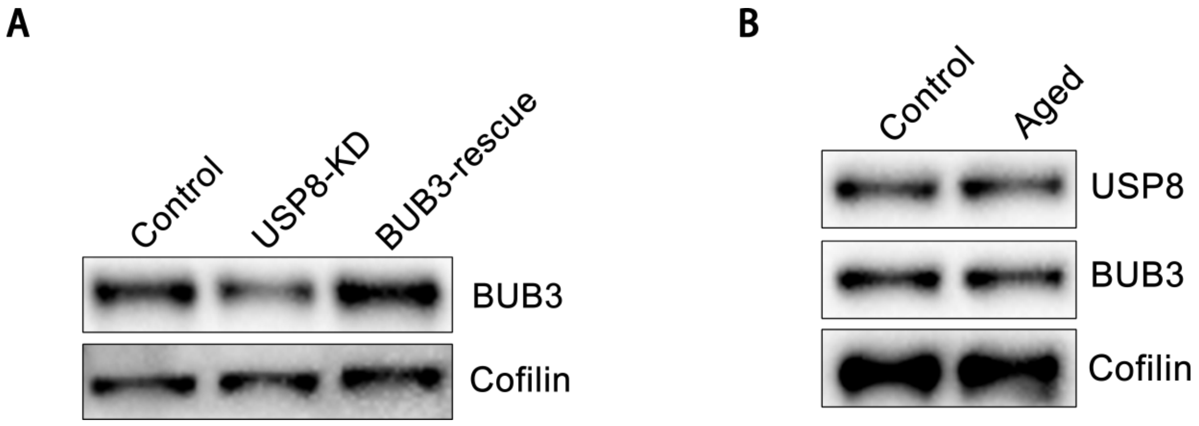

fig. S9. Detection of USP8 and/or BUB3 protein levels. (A) Protein levels of BUB3 were assessed by immunoblots in control, USP8-KD, and BUB3-rescue oocytes. The blots were probed with BUB3 and Cofilin antibodies. (B) Protein levels of USP8 and BUB3 were assessed by immunoblots in control and aged mouse oocytes (from 10-month-old female ICR mice). The blots were probed with USP8, BUB3, and Cofilin antibodies.

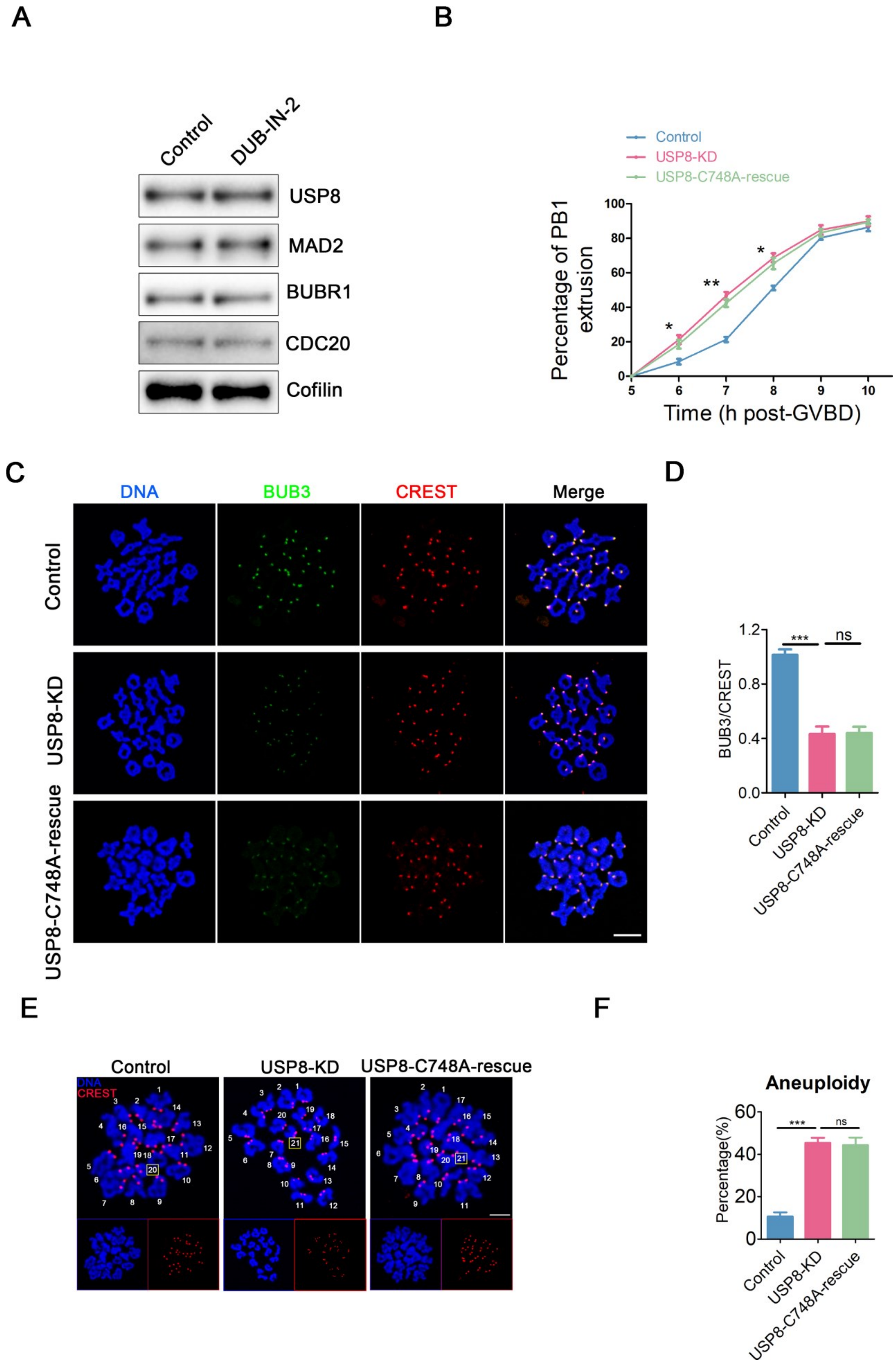
A
Control
DUB-IN-2
USP8
MAD2
BUBR1
CDC20
Cofilin
B
Control
USP8-KD
USP8-C748A-rescue
Percentage of PB1 extrusion
Time (h post-GVBD)
C
DNA
BUB3
CREST
Merge
Control
USP8-KD
USP8-C748A-rescue
D
BUB3/CREST
***
ns
Control
USP8-KD
USP8-C748A-rescue
E
Control
USP8-KD
USP8-C748A-rescue
DNA
CREST
F
Aneuploidy
Percentage(%)
***
ns
Control
USP8-KD
USP8-C748A-rescue

fig. S10. Effects of the loss of USP8 deubiquitinating activity on oocytes. (A) Immunoblotting analysis protein levels of USP8, MAD2, BUBR1, and CDC20 in control and DUB-IN-2 (20 μM)-treated oocytes. (B) Quantitative analysis of PBE rates were shown in control (n = 117), USP8-KD (n = 118), and USP8-C748A-rescue (n = 113) oocytes at consecutive time points of post-GVBD. (C) Localization of BUB3 at prometaphase I stage in control, USP8-KD, and USP8-C748A-rescue oocytes. At 3 hours after GVBD, oocytes were fixed and immunostained for BUB3, CREST and DNA (Hoechst). Scale bar, 10 μm. (D) The relative fluorescence intensities of BUB3 to CREST were measured in control (n = 200, kinetochores), USP8-KD (n = 200, kinetochores), and USP8-C748A-rescue (n = 200, kinetochores) oocytes. The signal intensity of BUB3 was normalized with that of CREST. (E) Representative images of euploid and aneuploid MII eggs. Chromosome spreading was performed to count the number of chromosomes in control, USP8-KD and USP8-C748A-rescue oocytes at 10 hours after GVBD. The total number of univalents is indicated by the yellow square. Scale bar, 7 μm. (F) The rates of aneuploid eggs were recorded in control (n = 30), USP8-KD (n = 31), and USP8-C748A-rescue (n = 27) oocytes. Data were presented as mean value (mean ± SEM) of at least three independent experiments. $^{*}P < 0.05$, $^{**}P < 0.01$, $^{***}P < 0.001$, ns, not significant.
